# TREM-1 triggers necroptosis of macrophages through mTOR-dependent mitochondrial fission during acute lung injury

**DOI:** 10.1186/s12967-023-04027-4

**Published:** 2023-03-06

**Authors:** Wen-Jing Zhong, Jun Zhang, Jia-Xi Duan, Chen-Yu Zhang, Sheng-Chao Ma, Yu-Sheng Li, Nan-Shi-Yu Yang, Hui-Hui Yang, Jian-Bing Xiong, Cha-Xiang Guan, Zhi-Xing Jiang, Zhi-Jian You, Yong Zhou

**Affiliations:** 1 Department of Physiology, School of Basic Medical Science, Central South University, Changsha, 410078 Hunan China; 2 Department of Physiology, Hunan University of Medicine, Huaihua, China; 3 NHC Key Laboratory of Metabolic Cardiovascular Diseases Research, Ningxia Medical University, Yinchuan, 750004 China; 4 The School of Basic Medical Sciences, Ningxia Medical University, Yinchuan, 750004 China; 5 Department of Orthopedics, Xiangya Hospital, Central South University, Changsha, 410008 Hunan China; 6 Department of Anesthesiology, Liuzhou People’s Hospital, Liuzhou, China; 7 Liuzhou Key Laboratory of Anesthesia and Brain Health, Liuzhou People’s Hospital, Liuzhou, China

**Keywords:** Acute lung injury, TREM-1, Macrophages, Necroptosis, Mitochondrial fission, mTOR

## Abstract

**Background:**

Necroptosis of macrophages is a necessary element in reinforcing intrapulmonary inflammation during acute lung injury (ALI). However, the molecular mechanism that sparks macrophage necroptosis is still unclear. Triggering receptor expressed on myeloid cells-1 (TREM-1) is a pattern recognition receptor expressed broadly on monocytes/macrophages. The influence of TREM-1 on the destiny of macrophages in ALI requires further investigation.

**Methods:**

TREM-1 decoy receptor LR12 was used to evaluate whether the TREM-1 activation induced necroptosis of macrophages in lipopolysaccharide (LPS)-induced ALI in mice. Then we used an agonist anti-TREM-1 Ab (Mab1187) to activate TREM-1 in vitro. Macrophages were treated with GSK872 (a RIPK3 inhibitor), Mdivi-1 (a DRP1 inhibitor), or Rapamycin (an mTOR inhibitor) to investigate whether TREM-1 could induce necroptosis in macrophages, and the mechanism of this process.

**Results:**

We first observed that the blockade of TREM-1 attenuated alveolar macrophage (AlvMs) necroptosis in mice with LPS-induced ALI. In vitro, TREM-1 activation induced necroptosis of macrophages. mTOR has been previously linked to macrophage polarization and migration. We discovered that mTOR had a previously unrecognized function in modulating TREM-1-mediated mitochondrial fission, mitophagy, and necroptosis. Moreover, TREM-1 activation promoted DRP1^Ser616^ phosphorylation through mTOR signaling, which in turn caused surplus mitochondrial fission-mediated necroptosis of macrophages, consequently exacerbating ALI.

**Conclusion:**

In this study, we reported that TREM-1 acted as a necroptotic stimulus of AlvMs, fueling inflammation and aggravating ALI. We also provided compelling evidence suggesting that mTOR-dependent mitochondrial fission is the underpinning of TREM-1-triggered necroptosis and inflammation. Therefore, regulation of necroptosis by targeting TREM-1 may provide a new therapeutic target for ALI in the future.

**Supplementary Information:**

The online version contains supplementary material available at 10.1186/s12967-023-04027-4.

## Introduction

Acute lung injury (ALI), a progressive, life-threatening clinical condition, is characterized by local inflammatory accumulation, an elevated penetrability of the alveolar-capillary barrier, denudation of the alveolar epithelium, and pulmonary edema [1]. The pro-inflammatory cascade response is particularly significant in the pathogenesis of ALI [2]. Alveolar macrophages (AlvMs) account for approximately 95% of the airspace leukocytes [3]. AlvMs occupy a vital position in initiating ALI through synthesizing and releasing various inflammatory mediators [4]. Our previous studies have found that depleted macrophages significantly attenuate lung tissue pathological injury [5]. For this reason, their response to an infection can critically influence the outcomes of infectious diseases. Studies have demonstrated that a variety of stimuli, such as viral, bacterial, or mechanical traction, can lead to AlvM death [6, 7]. Viral, bacterial, or physical stimuli can result in AlvM death [6, 7]. On the one hand, these dead macrophages allow invading bacterial viruses to escape the surveillance of intrinsic immunity and contribute to the expansion and multiplication of bacterial viruses [8]. On the other hand, these cell deaths release cell debris or damage-associated molecular patterns (DAMPs) that activate other immune cell populations in the lung, forming a positive feedback loop, which amplifies the inflammatory cascade response and exacerbates ALI [9]. There is increasing recognition that inflammation and macrophage death are inextricably linked, forming a self-perpetuating cycle that exaggerates inflammation [9].

Necroptosis is recently thought of as a novel mechanism contributing to the inflammatory cascade response to ALI [7, 10]. Necroptosis occurs through the activity of the necroptosome, a complex of two essential proteins: receptor-interacting serine/threonine-protein kinase 3 (RIPK3) and mixed lineage kinase domain-like pseudokinase (MLKL) [11]. RIPK3 phosphorylates and recruits MLKL into the necroptosome. Then phosphorylated MLKL oligomerizes and translocates to the plasma membrane to create pores for osmotic rupture [12]. Necroptosis results in the disrupture of the cellular membrane and the release of components, including DAMPs and inflammatory cytokines such as tumor necrosis factor-alpha (TNF-α), which can cause a self-amplified inflammatory process [13]. Our recent studies found that L‐OPA1 deficiency and mitochondrial citrate accumulation mediate mitochondrial fission, induce necroptosis of alveolar epithelial cells, and exacerbate ALI [10, 14]. For macrophages, the human respiratory syncytial virus (RSV) induces necroptosis of macrophages, aggravating pneumonia [15]. *Ripk3*^−/−^ mice were protected from RSV-induced weight loss and presented with reduced viral loads in the lungs [7]. Nonetheless, the molecular mechanism that triggers macrophage necroptosis during ALI is not fully understood.

Triggering receptor expressed on myeloid cells-1 (TREM-1) is a pattern recognition receptor expressed broadly on monocytes/macrophages and neutrophils [16]. Studies from others [17] and us [18, 19] strongly suggest that TREM-1 activation orchestrates the monocyte/macrophage pro-inflammatory responses. Engagement of TREM-1, after association with the adaptor protein 12-kDa DNAX activating protein (DAP12), has been shown to stimulate the production of pro-inflammatory cytokines and chemokines [20]. TREM-1 is a critical orchestrator of the inflammatory response in ALI. Our previous studies reveal that blocking TREM-1 ameliorates lipopolysaccharide (LPS)-induced ALI [18, 19]. Peptides from the N-terminal fragment of Tag7 block TREM-1, inhibiting the production of pro-inflammatory cytokines in the murine model of ALI with diffuse alveolar damage [21]. However, the role of TREM-1 in macrophage fate during ALI needs further investigation.

Mitochondrial dynamics is essential for the survival and function of eukaryotic organisms [22]. Mitochondria are in a highly dynamic meshwork structure, constantly in the process of fission and fusion, known as mitochondrial dynamics [23]. Mitochondrial dynamics equilibrium is disrupted upon the stimulation of DAMPs or pathogen-associated molecular patterns (PAMPs). Excessive mitochondrial fission leads to persistent mitochondrial loss, triggering cell death [24]. Mitochondrial fission mainly depends on GTPase dynamin-related protein 1 (DRP1), a member of the dynamin family of GTPases [25]. Phosphorylation of DRP1 at Ser616 encourages its recruitment to the outer mitochondrial membrane to drive scission [26]. The nutrient-sensing mechanistic/mammalian target of rapamycin complex (mTOR) reportedly stimulates DRP1-mediated mitochondrial fission via 4E-BP-mediated translational regulation [27]. DRP1 and the retinoblastoma protein interact to mediate mitochondria-dependent necroptosis induced by cadmium in hepatocytes [28]. While inhibiting mitochondrial fission protects against cigarette smoke-induced mitochondrial dysfunction and necroptosis in alveolar epithelial cells [29]. What’s more, DRP1-mediated mitochondrial fission is necessary for mitophagy, a “self-eating” process [30]. Excessive mitophagy is maladaptive and associated with cell death [31]. Recently, we have reported that excessive mitochondrial fission induced by mitochondrial citrate accumulation contributes to necroptosis by triggering mitophagy in alveolar epithelial cells [14]. Thus, mitochondrial fission would be a key mechanism triggering necroptosis.

In this study, we first observed that the blockade of TREM-1 attenuated necroptosis of AlvMs in LPS-induced ALI in mice. In vitro, TREM-1 activation induced necroptosis of macrophages. Mechanistically, we found that TREM-1 promoted DRP1-dependent mitochondrial fission via mTOR signaling, which exacerbated mitophagy, resulting in necroptosis of macrophages. The purpose of our study was to find the target that triggers the necroptosis of macrophages during ALI. And we found that targeting TREM-1-triggered necroptosis may provide a new therapeutic pathway for ALI and other inflammatory diseases.

## Materials and methods

### Mice and induction of ALI

Male C57BL/6 J mice (22 ± 2 g; from the Hunan SJA Laboratory Animal Co., Ltd., Hunan, China) were used in this study. All experiments were approved by the IRB of the school of Basic Medical Science at Central South University (202110096, Changsha, China). Mice were anesthetized with pentobarbital sodium and intratracheally injected with LPS (5 mg/kg, from E. coli O111:B4, Sigma-Aldrich, MO, USA). Control mice were injected with 50 μL saline. The antagonistic TREM-1 peptide (LR12, LQEEDAGEYGCV, 5 mg/kg) or a sequence-scrambled negative control peptide (LRS, YQVGELCTGEED, 5 mg/kg), according to our previous studies [18], was intravenously injected 2 h before the LPS administration. The dodecapeptide was chemically synthesized (GL Biochem, China) as COOH terminally amidated peptides with > 95% purity, as confirmed by mass spectrometry and analytical reverse-phase high-performance liquid chromatography. All mice were sacrificed 12 h after the LPS injection.

### Hematoxylin and eosin (H&E)

Twelve hours after the LPS injection, the lungs of the mice were inflated and fixed with 4% neutral buffered formaldehyde solution. Multiple Sects. (4 µm) were sliced and stained with H&E (Solarbio, China, Beijing). Images were taken with Pannoramic Scan (3Dhistech, Hungary, Budapest).

### Flow cytometry

Single-cell suspensions of the lung tissue were prepared for flow cytometry. In brief, lung tissue was incubated in collagenase I (1 mg/mL, Roche, Mannheim, Germany) at 37 °C with frequent agitation for 45 min. After all dissociation procedures, cells were washed with a dissociation medium, filtered through a 70-mesh and 40-mesh cell strainer, and centrifuged at 1200 rpm for 10 min. Cells were suspended in an ice-cold phosphate-buffered saline (PBS) buffer. Samples were stained using the following antibodies: Fixable viability stain 450, PE-conjugated CD64 (FcγRI), APC-Cy7-conjugated CD45 (30-F11), APC-conjugated CD11b, BV510-conjugated Siglec-F, PE/Cyanine7-conjugated F4-80, Brilliant violet 605-conjugated CD11c and CoraLite®488-conjugated MLKL monoclonal antibody. The information of those antibodies is shown in Table 1. The cells were washed with PBS, and the cytometry buffer was added. The cells were subjected to analysis using a BD FACS Verse (BD Biosciences, San Jose, CA, USA) flow cytometer and analyzed using FlowJo software (version 10, Tree Star Inc., San Jose, CA, USA).

**Table 1:** Antibody sources and dilutions

Antibody	Source	Catalog	Dilution ratio
*Primary antibodies for Western blotting*			
Rabbit anti-MLKL polyclonal antibody	Abcam	Ab172868	1:2000
Rabbit anti-phospho-MLKL phospho-S345 monoclonal antibody	Abcam	Ab196436	1:2000
Rabbit-anti-RIPK3 polyclonal antibody	Abcam	Ab62344	1:2000
Rabbit anti-phospho-RIPK3 phospho-S232 monoclonal antibody	Abcam	Ab195117	1:2000
Anti-IL-1β polyclonal antibody	R&D	AF-401-NA	1:2000
Anti-DRP1 antibody	Abcam	Ab184247	1:2000
Anti-p-DRP1 (phospho S616) antibody	CST	3455	1:2000
Rabbit-anti-TOM20 antibody	Proteintech	11802-1-AP	1:2000
Mouse-anti-OPA1 antibody	BD	612606	1:2000
Rabbit-anti-MFN2 antibody	Proteintech	12186-1-AP	1:2000
Rabbit-anti-MTFP1 antibody	Proteintech	14257-1-AP	1:1000
Rabbit-anti-PGAM5 antibody	Proteintech	28,445–1-AP	1:3000
Anti-BAX antibody	CST	2772	1:2000
Rabbit-anti-BCL2 antibody	Cohesion	CPA1095	1:1000
Rabbit-anti-caspase3 antibody	CST	9662	1:2000
Rabbit-anti-caspase6 antibody	CST	9762	1:2000
GSDMDC1 Antibody (H-11)	Santa Cruz	sc-393581	1:2000
Rabbit-anti-LC3A/B(D3U4C) antibody	CST	12741	1:2000
Rabbit-anti-Pink1 antibody	Abcam	Ab23707	1:1000
Rabbit-anti-beclin1 antibody	CST	3738	1:2000
Anti-mTOR monoclonal antibody	Proteintech	66888-1-lg	1:2000
Anti-phospho-mTOR (Ser2448) Antibody	Proteintech	67778-1-lg	1:2000
Rabbit-anti-EIF4E antibody	Proteintech	11149-1-AP	1:2000
Rabbit-anti-P-EIF4EBP1-S65 antibody	Proteintech	#12721	1:2000
Anti-α-tubulin monoclonal antibody	Servicebio	GB11200	1:10,000
*Secondary antibodies for Western blotting*			
HRP-conjugated goat anti-rabbit IgG	SAB	#L3012-2	1: 5000
Goat anti-Mouse IgG	SAB	L3032	1: 5000
*Primary antibodies for immunofluorescence*			
Rabbit anti-TOM20 polyclonal antibody	Proteintech	11802-1-AP	1: 200
CoraLite®488-conjugated MLKL antibody	Proteintech	CL488-66675	1: 300
*Antibodies for Flow Cytometry*			
PE-conjugated CD64 (FcγRI)	BioLegend	139304	1:100
Fixable Viability Stain 450	BioLegend	562247	1:1000
APC-Cy7-conjugated CD45 (30-F11)	BioLegend	557659	1:100
BV510-conjugated Siglec-F	BioLegend	740158	1:100
APC-conjugated CD11b	BioLegend	101212	1:100
Brilliant Violet 605-conjugated CD11c	BioLegend	301636	1:100
PE/Cyanine7-conjugated F4-80	BioLegend	123114	1:100
CoraLite®488-conjugated MLKL antibody	Proteintech	CL488-66675	1: 200

### Isolation and culture of primary murine peritoneal macrophages

Primary murine peritoneal macrophages were isolated according to our previous studies [32, 33]. C57BL/6 J mice (22 ± 2 g) were intraperitoneally injected with 3 mL of thioglycollate (Sigma-Aldrich). Three days later, the peritoneal lavage solution was isolated. After lysis of red blood cells and washing with PBS solution, macrophages were cultured and plated into 24-well plates (0.5 × 10^6^ cells/well) in RPMI-1640 (Gibco, Life Technologies, Carlsbad, CA) with 10% heat-inactivated bovine calf serum (BCS, Gibco) at 37 °C. Peritoneal macrophages were enriched by plastic adherence for 1.5–2 h. The non-adherent cells were removed by washing with warm PBS, and the adhesive cells were cultured for further experiments.

### Treatments of macrophages

To activate TREM-1 in vitro, we coated 24-well plates with the agonist anti-TREM-1 mAbs (10 μg/mL, Mab1187, R&D Systems, USA) at 37 ℃ overnight and washed twice with PBS. Then purified macrophages (0.5 × 10^6^ cells/well in RPMI 1640) or macrophages pre-treated with the RIPK3 inhibitor (GSK872, 1 μM, Medchem Express, USA), the DRP1 inhibitor (Mdivi-1, 100 nM, Medchem Express) or the mTOR inhibitor (Rapamycin, 100 nM, Beyotime, Jiangsu, China) were added. Cells were cultured for 12 h or 24 h and harvested for gene or protein detection.

### Cell counting kit-8 assay

Macrophages were cultured into 96-well plates (1000 cells/well). Cell survival rate was determined with a cell counting kit‐8 (CCK‐8) (TargetMol, China). The culture was added to CCK‐8 solution and incubated for 2 h at 37 °C with 5% CO_2_. The absorbance was measured at 450 nm using a microplate reader (Miulab, Hangzhou, China).

### Western blot analysis

Macrophages were harvested, and proteins were extracted using RIPA buffer (Beyotime) containing protease inhibitors PMSF (Servicebio, China) and cocktail (Roche). For the protein preparation of the culture supernatants, 700 μL culture supernatants were collected and mixed with 700 μL 100% methanol and 175 μL trichloromethane. Supernatants were centrifuged at 13,000 rpm for 5 min at 4 °C to precipitate the proteins. The remaining proteins were resuspended in 20 μL10% SDS. 30 µg of cell lysate samples or culture supernatant samples were resolved by a 12% SDS/PAGE. Western blotting was performed as described previously [34]. The blots were visualized by ChemiDoc XRS (Bio-Rad, USA). The relative band intensity was quantified using the Image Lab Analyzer software (Bio-Rad). α-tubulin was used as the loading control. The antibodies used in the study are shown in Table 1.

### Real-time PCR

RNA was isolated using RNAisol (TaKaRa Clontech, Japan) as described [35]. Reverse transcription with 1 μg of RNA was carried out in a T100TM Thermal Cycler (Bio-Rad) using a high-capacity cDNA reverse transcription kit (TaKaRa Clontech). Amplification was performed by quantitative real‐time PCR (qPCR) in a Bio-Rad real-time PCR system (CFX96 Touch™; Bio-Rad) with SYBR® Premix Ex Taq™ II system (TaKaRa Clontech). Relative gene expression was analyzed using the 2^–ΔΔCt^ method. The primer sequences of the targeted gene used in the study are shown in Table 2.

**Table 2:** Sequences of the primers used in this study

Gene	Forward primer (5’-3’)	Reverse primer (5’-3’)
*Dnm1*	AATATGCCGAGTTCCTGCACT	GTCTCAGCCTCGATCTCCAG
*Mff1*	CACCACCAAATGCTGACCTG	GGTGTTTTCAGTGCCAGAGG
*Mtfp1*	TAATCCACCCCATCGACAG	TCCACTGACGGGTACAGCTT
*Mfn1*	CCTACTGCTCCTTCTAACCCA	AGGGACGCCAATCCTGTGA
*Mfn2*	CGGTTCACTGTACCCCACTT	GAGGCCAGTAGTGTTGCCTT
*Opa1*	GATGACACGCTCTCCAGTGA	TCGGGGCTAACAGTACAACC
*β-actin*	TTCCAGCCTTCCTTCTTG	GGAGCCAGAGCAGTAATC

### Cytokine detection

TNF-α contents in the cell culture supernatant were measured using appropriate enzyme-linked immunosorbent assay (ELISA) kits according to the manufacturer’s protocols (Invitrogen, Thermo Fisher Scientific, USA).

### Immunofluorescence

Macrophages were stimulated with Mab1187 (10 μg/mL) for 24 h and then washed with PBS twice for 5 min, fixed with 4% paraformaldehyde for 15 min at room temperature in order to image mitochondrial morphology. The cells were incubated with 0.1% Triton X-100, blocked in 1% BSA for 30 min before being stained with an anti-rabbit TOM20 antibody (1:200, Proteintech, Wuhan, China) or a CoraLite®488-conjugated MLKL monoclonal antibody (1:250, Proteintech) at 4 °C overnight. After washing 3 times with PBS, the cells were incubated with FITC goat anti-rabbit IgG (ABclonal, China) for 1 h at room temperature. The nuclei were counterstained with DAPI for 1 min (Solarbio, China). Images were acquired with a Laser Scanning Confocal Microscope (Leica SP8, Germany).

Lung slides were placed in 0.01 M citrate buffer (pH 6.0). Heat citrate buffers until it boils and keeps it boiling for 10 min. Then the slides were washed with PBS thrice for 5 min and then blocked with 5% BSA for 1 h. Subsequently, sections were incubated with primary antibodies, including a CoraLite®488-conjugated MLKL monoclonal antibody (1:500, Proteintech) and anti-F4-80 (1:200). Next, similar fluorescence experiments were used for the lung sections. Images were taken with a Pannoramic Scan (3Dhistech, Hungary, Budapest).

### Measurement of mitochondrial membrane potential

According to the manufacturer's protocols, the mitochondrial membrane potential was measured using the JC-1 Assay Kit (Beyotime, Shanghai, China). In brief, macrophages were stimulated with Mab1187 (10 μg/mL) for 24 h and then washed with PBS. The cells were incubated with a JC-1 working solution (1640 medium: JC-1 working solution = 1:1) in the dark at 37℃ for 20 min. Images were obtained using a fluorescence microscope. The red/green immunosignals were analyzed by Image J software.

### Statistical analysis

N represents experiments performed on individual mice or different macrophages from separate mice. All experiments in this study were independently repeated at least three times. Data were shown as the mean ± SD values and analyzed with GraphPad Prism 7 software (San Diego, CA, USA) and SPSS version 23.0 (SPSS, Chicago, IL, USA). All data follow a normal distribution, tested with the Shapiro–Wilk test. Statistical comparisons between two-group were determined by unpaired *t*-test. Differences among multiple groups were determined by ANOVA, followed by the Bonferroni correction for multiple comparison testing. *P* < 0.05 was regarded as statistically significant.

## Results

### Pharmacologic blockade of TREM-1 attenuates macrophage necroptosis in LPS-induced ALI mice

**Fig. 1:**
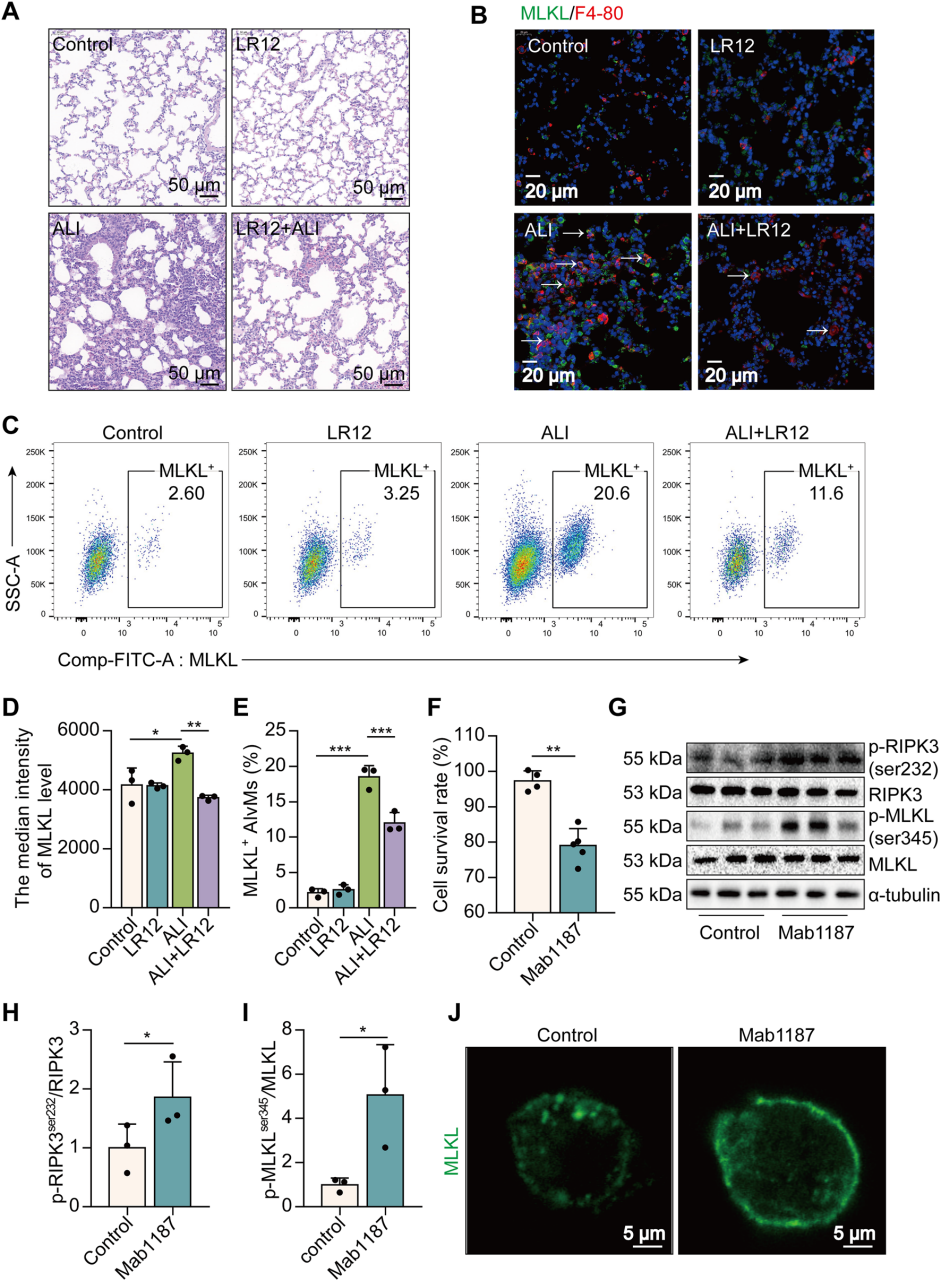
Blockade of TREM-1 reduces macrophage necroptosis in LPS-induced ALI mice. **A** H&E staining. **B** the localization of MLKL (green) and F4-80 (red). **C**–**E** flow cytometry analysis of MLKL^+^ AlvMs. **F**, CCK‐8. **G**–**I** p-RIPK3^ser232^, RIPK3, p-MLKL^ser345^, and MLKL protein. **J** MLKL (green) expression

To evaluate if TREM-1 activation leads to necroptosis of macrophages, thereby aggravating ALI, we initially exposed mice to the LR12 TREM-1 decoy receptor, which has been validated in rodents [17, 36]. Histological study showed that LR12-treated mice lungs had less leukocyte infiltration, alveolar congestion, and alveolar barrier thickening than LPS-treated lungs (Fig. 1A). Interestingly, a large number of F4-80 (red) and MLKL (green) proteins were co‐expressed in the lungs of ALI mice, while LR12 treatment significantly decreased the MLKL^+^ macrophages (Fig. 1B). We next assessed whether MLKL is expressed in particular macrophage subtypes like alveolar macrophages (AlvMs) or interstitial macrophages (IntMs). Lymphocyte common antigen (CD45) is a receptor-linked protein tyrosine phosphatase expressed on all leucocytes. Fc gamma receptor I (FcγRI, CD64) is a well-known antigen for monocytes/macrophages/neutrophils [37]. AlvMs express low levels of CD11b but have high levels of expression of CD11c and Siglec-F [3]. CD11b^hi^ Siglec-F^lo^ macrophages that occupy the lung interstitium. So we used F4-80, CD64, Siglec-F, CD11b, and CD11c as markers that distinguish AlvMs from IntMs [38]. A statistically significant decrease in MLKL^+^ AlvMs (CD45^+^ CD64^+^ F4-80^+^ CD11c^+^ Siglec-F^+^ MLKL^+^cells) was found in the lungs of LR12-treated mice compared with those of ALI-treated mice (Fig. 1C). The blockade of TREM-1 reduced the median intensity of MLKL and the percentage of positive cells in AlvMs of ALI mice (Fig. 1D, E). However, no altered levels of MLKL were found in IntMs (CD45^+^ CD64^+^ F4-80^+^ CD11b^+^ Siglec-F^−^) (Additional file 1: Fig. S1A–D). To further determine the effects of TREM-1 activation on the necroptosis of macrophages, we used an agonist anti-TREM-1 Ab (Mab1187) at the cellular level [39]. TREM-1 activation reduced the viability of macrophages (Fig. 1F). Notably, the necroptosis-related proteins RIPK3^ser232^ and MLKL^ser345^ phosphorylation were highly induced in macrophages pre-treated with Mab1187 (Fig. 1G–I). TREM-1 activation also induced translocation of the trimerized MLKL to the plasma membrane to form pores, causing macrophages to swell (Fig. 1J). Together, these data reveal an unexpected link between TREM-1 activation and macrophage necroptosis in ALI.

### RIPK3 kinase activity is essential for TREM-1 activation-induced necroptosis in macrophages

**Fig. 2:**
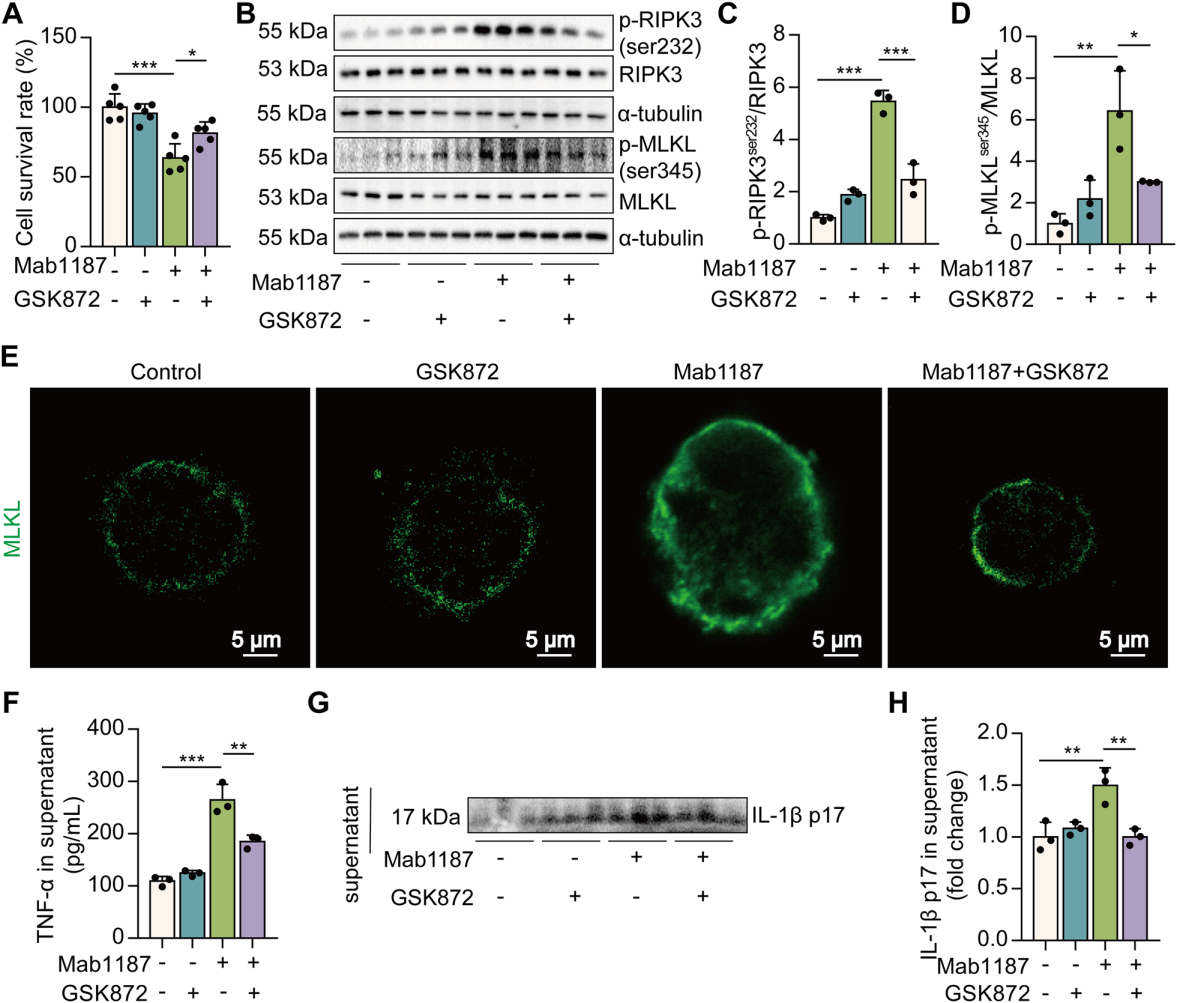
TREM-1 activation triggers macrophage necroptosis dependently on RIPK3. **A** The cell survival rate was examined by CCK‐8, *n* = 5. **B**–**D** p-RIPK3^ser232^, RIPK3, p-MLKL^ser345^, and MLKL protein, *n* = 3. **E** Immunofluorescence staining for MLKL (green); scale bars = 5 μm. **F** TNF‐α production in the supernatant. **G**, **H** IL-1β p17 level in the supernatant

To investigate the role of RIPK3 in TREM-1-mediated macrophage necroptosis, we pre-treated macrophages with GSK872, an inhibitor of RIPK3. Pre-treating macrophages with GSK872 significantly attenuated cell death by TREM-1 activation (Fig. 2A). GSK872 also alleviated the phosphorylation of necroptosis-related proteins, RIPK3 and MLKL, induced by TREM-1 activation (Fig. 2B–D). RIPK3 inhibitor reduced TREM-1-induced trimerization of MLKL, which translocated to the plasma membrane to form pores (Fig. 2E). Necroptosis of macrophage leads to the release of cytosolic inflammatory cytokine contents [40]. Next, we elucidated the role of necroptosis pathways on TNF-α and IL-1β release induced by TREM-1 activation. GSK872 significantly reduced TNF-α and IL-1β p17 secretion, which was promoted by TREM-1 activation in macrophages (Fig. 2F-H), suggesting that necroptosis pathways are necessary for TREM-1-elicited inflammatory factor release. Collectively, these data illustrate that TREM-1 activation results in RIPK3 kinase activity-dependent necroptosis in macrophages.

### TREM-1 activation triggers mitochondrial fission in macrophages

**Fig. 3:**
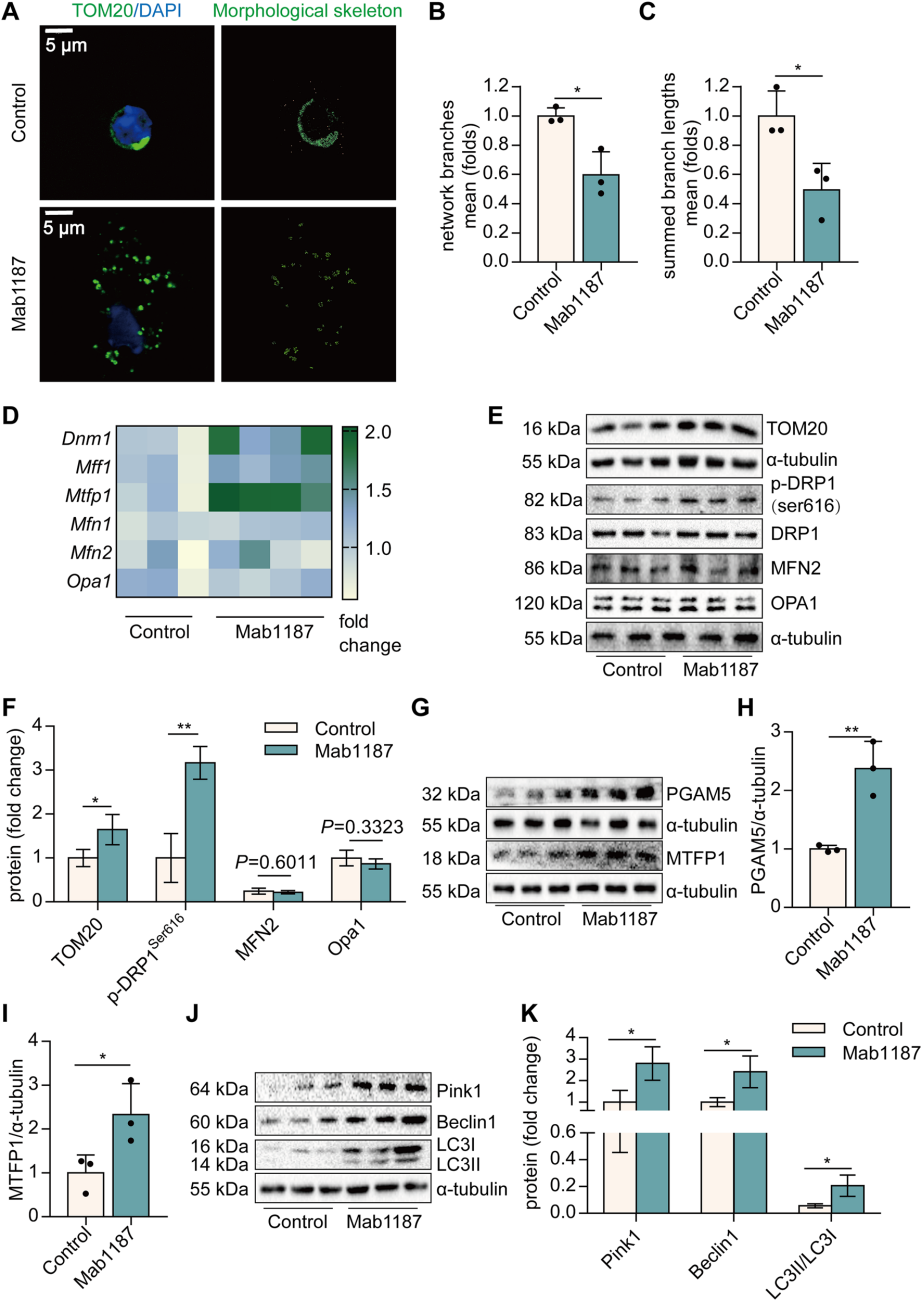
TREM-1 activation promotes mitochondrial fragmentation and mitophagy of macrophages. **A** Mitochondrial morphology stained with TOM20. **B**–**C** The morphological skeleton and quantification of mitochondrial morphology. **D** *Dnm1*, *Mff1*, *Mtfp1*, *Mfn1*, *Mfn2,* and *Opa1* mRNA. **E**–**F** Protein levels of TOM20, DRP1, MFN2 and OPA1. **G**–**I** PGAM5, and MTFP1 protein. **J-K** Pink1, Beclin1, and LC3 protein

Mitochondria play a pivotal role in necroptosis. Mitochondrial membrane potential, an index of mitochondrial function, was dissipated by the treatment with Mab1187 (Additional file 1: Fig. S2A, B), indicating that TREM-1 activation exacerbates mitochondrial dysfunction of macrophages. We then characterized the morphology of the mitochondrial network by immunocytochemistry, using an antibody against the outer mitochondrial membrane protein TOM20. A significant fragmentation was found in TREM-1-activated macrophages (Fig. 3A–C). TOM20 protein expression was also increased in TREM-1-activated macrophages (Fig. 3E, F). We measured the levels of several proteins involved in mitochondrial fission and fusion by real-time PCR and western blot analysis. The mitochondrial outer membrane fusion is mediated by mitofusin (MFN), and the mitochondrial inner membrane fusion is oriented by optic atrophy protein 1 (OPA1) in mammals. We found no difference in the levels of the mitochondrial fusion proteins OPA1 or MFN2 between wild-type and TREM-1-activated macrophages (Fig. 3E, F). Mitochondrial fission relies on DRP1 and its mitochondrial anchor’s mitochondrial fission factor (MFF). The phosphorylation of DRP1^s616^ promotes mitochondrial localization. We found that TREM-1 activation significantly increased *Dnm1* (DRP1 gene) mRNA and p-DRP1^s616^ levels (Fig. 3D–F). In addition, *Mff* mRNA was also increased in TREM-1-activated macrophages (Fig. 3D). Mitochondrial fission process protein 1 (MTFP1) and phosphatase phosphoglycerate mutase family member 5 (PGAM5) are coupled to the mitochondrial recruitment and activation of DRP1. TREM-1 activation upregulated the expression of MTFP1 and PGAM5 protein (Fig. 3G–I). These results suggest that activation of TREM-1 induced mitochondrial fission in macrophages. Excessively fragmented mitochondria were eliminated through mitochondria mitophagy [41]. The phosphatase and tensin homolog-induced kinase 1 (Pink1), Beclin1, and LC3II/LC3I ratio of mitophagy-related protein were upregulated in TREM-1-activated macrophages (Fig. 3J, K). Treatment with Mdivi-1, a selective DRP1 inhibitor, simultaneously reduced TREM-1-induced mitophagy (Additional file 1: Fig. S3A–D). These results suggest that TREM-1 activation induces mitochondrial fission and subsequent mitophagy in macrophages.

### Blockade of DRP1 reverses TREM-1-induced necroptosis in macrophages

**Fig. 4:**
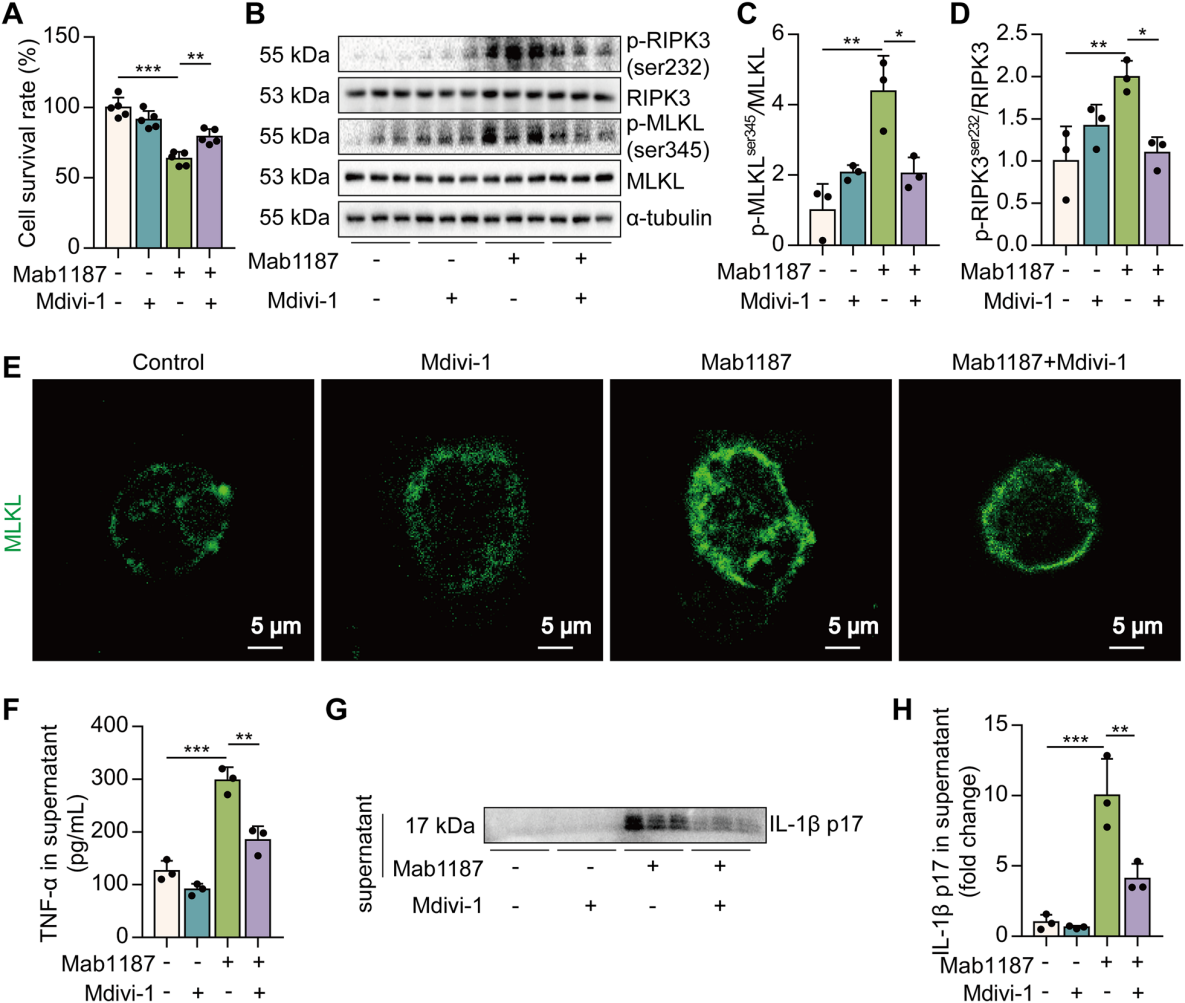
TREM-1 activation induces necroptosis through mitochondrial fission in macrophages. **A** The cell survival rate, *n* = 5. **B**–**D** Protein levels of RIPK3, MLKL, and their phosphorylation, *n* = 3. **E** Immunofluorescence staining for MLKL (green); scale bars = 5 μm. **F** The concentration of TNF‐α in the supernatant, *n* = 3. **G**, **H** The level of IL-1β p17 in macrophages supernatant, *n* = 3 biological replicates

To assess the role of mitochondrial fission in TREM-1-mediated necroptosis, we used Mdivi-1, an inhibitor of DRP1. First, Mdivi-1 suppressed cell death induced by TREM-1 activation (Fig. 4A). Then, we observed that Mdivi-1 reduced the phosphorylation of RIPK3 and MLKL (Fig. 4B–D). Mdivi-1 also reduced the translocation of trimerized MLKL to the plasma membrane to form pores induced by TREM-1 activation (Fig. 4E). The pharmacological inhibition of DRP1 significantly reduced TNF-α and IL-1β p17 secretion promoted by TREM-1 activation in macrophages (Fig. 4F–H). In addition to necroptosis, we have focused on several classic forms of programmed cell death, including apoptosis and pyroptosis. We found that TREM-1 activation induced the pro-apoptotic protein caspase-6, caspase-3, and Bax and down-regulated the anti-apoptotic protein BCL2, suggesting that TREM-1 activation-induced apoptosis of macrophages. However, the pharmacological inhibition of DRP1 failed to reverse TREM-1-induced apoptosis in macrophages (Additional file 1: Fig. S4A–D). There is no difference in the level of the pyroptotic protein gasdermin D (GSDMD) between wild-type and TREM-1-activated macrophages (Additional file 1: Fig. S4E–G). Collectively, these data indicate that excessive mitochondrial fission is necessary for TREM-1-induced necroptosis in macrophages.

### TREM-1 instigates mitochondrial fission in an mTOR-dependent manner in macrophages

**Fig. 5:**
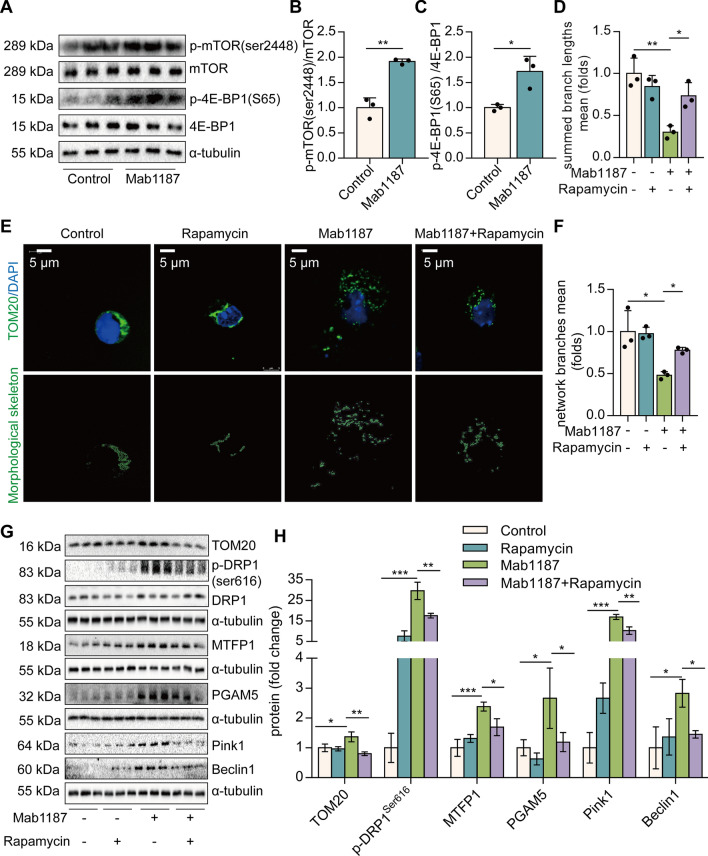
mTOR regulates TREM-1-induced mitochondrial fission in macrophages. **A**–**C** Protein levels of p-mTOR^ser2448^ and p-4E-BP1^S65^, *n* = 3. **D**-**E**, mitochondrial morphology was stained with TOM20 (green) and the morphological skeleton. **F**, quantification of mitochondrial morphology, *n* = 3. **G**, **H** Mitochondrial fission-related proteins: TOM20, DRP1^Ser616^, MTFP1, and PGAM5 proteins, *n* = 3. **G**, **H** Mitophagy-related proteins: Pink1 and Beclin1, *n* = 3

We next investigated how TREM-1 activation induces mitochondrial fission in macrophages. mTOR is reportedly to control mitochondrial fission [27]. We found that TREM-1-activated macrophages expressed significantly higher levels of translation initiation factor 4E (eIF4E)-binding protein1 (4E-BP1) and mTOR^ser2448^ phosphorylation (Fig. 5A–C). Inhibition of mTOR with rapamycin showed a partial restoration in mitochondrial membrane potential induced by anti-TREM-1 Ab (Additional file 1: Fig. S5A, B). We then characterized the morphology of the mitochondrial network. Inhibition of mTOR showed a reduction in mitochondrial fragmentation in TREM-1-activated macrophages (Fig. 5D–F). In addition, we observed that inhibition of mTOR decreased the levels of TOM20, p-DRP1^s616^, and PGAM5 (Fig. 5G, H). MTFP1 is an integral protein of the mitochondrial intermembrane whose overexpression engenders fragmentation [42]. Protein expression of MTFP1 induced by TREM-1 activation was also decreased by Rapamycin in macrophages (Fig. 5G, H). Besides, rapamycin also decreased the expression of mitophagy-related proteins Pink1 and Beclin1, induced by TREM-1 activation (Fig. 5G, H). All of these data imply that TREM-1 induces mitochondrial fission in an mTOR-dependent manner.

### Activation of TREM-1 induces necroptosis via mTOR in macrophages

**Fig. 6:**
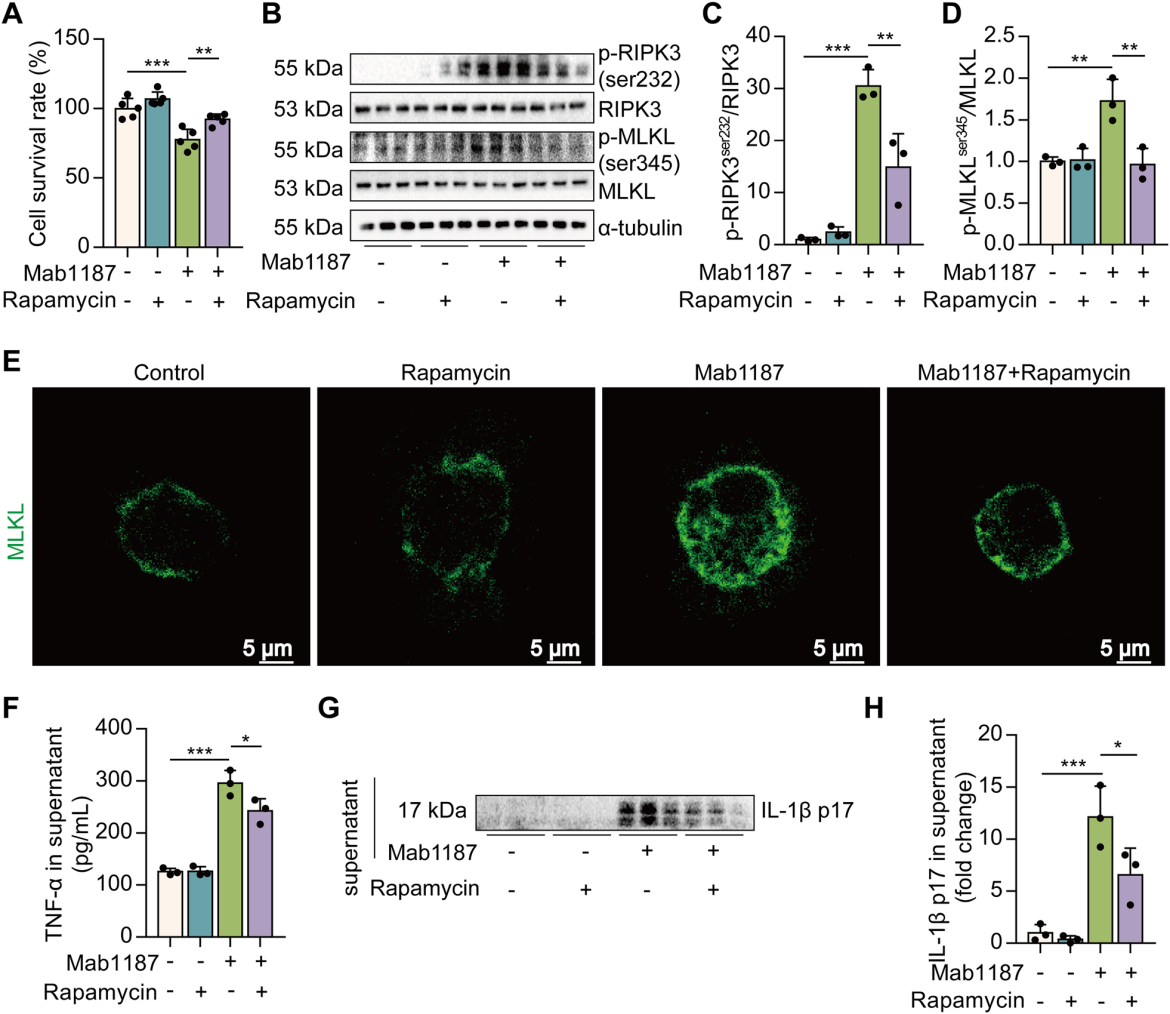
mTOR regulates TREM-1-induced necroptosis of macrophages. **A** the cell survival rate, *n* = 5. **B**–**D** Protein expression of p-RIPK3^ser232^, RIPK3, p-MLKL^ser345^, and MLKL, *n* = 3. **E** Immunofluorescence staining for MLKL (green) expression; scale bars = 5 μm. **F** The concentration of TNF‐α was assayed using ELISA, *n* = 3. **G**, **H** IL-1β p17 level in the supernatant, *n* = 3 biological replicates

Finally, we investigated whether TREM-1 induces necroptosis through mTOR signaling. Pre-treating macrophages with rapamycin significantly attenuated cell death by TREM-1 activation (Fig. 6A). mTOR inhibitor alleviated the phosphorylation of RIPK3 and MLKL induced by TREM-1 activation (Fig. 6B–D). Rapamycin reduced TREM-1-induced translocation of trimerized MLKL to the plasma membrane (Fig. 6E). Next, we found that rapamycin significantly reduced TNF-α and IL-1β release promoted by TREM-1 activation in macrophages (Fig. 6F–H). These results point to the critical role of the mTOR signal in the process of TREM-1-induced necroptosis in macrophages.

## Discussion

**Fig. 7:**
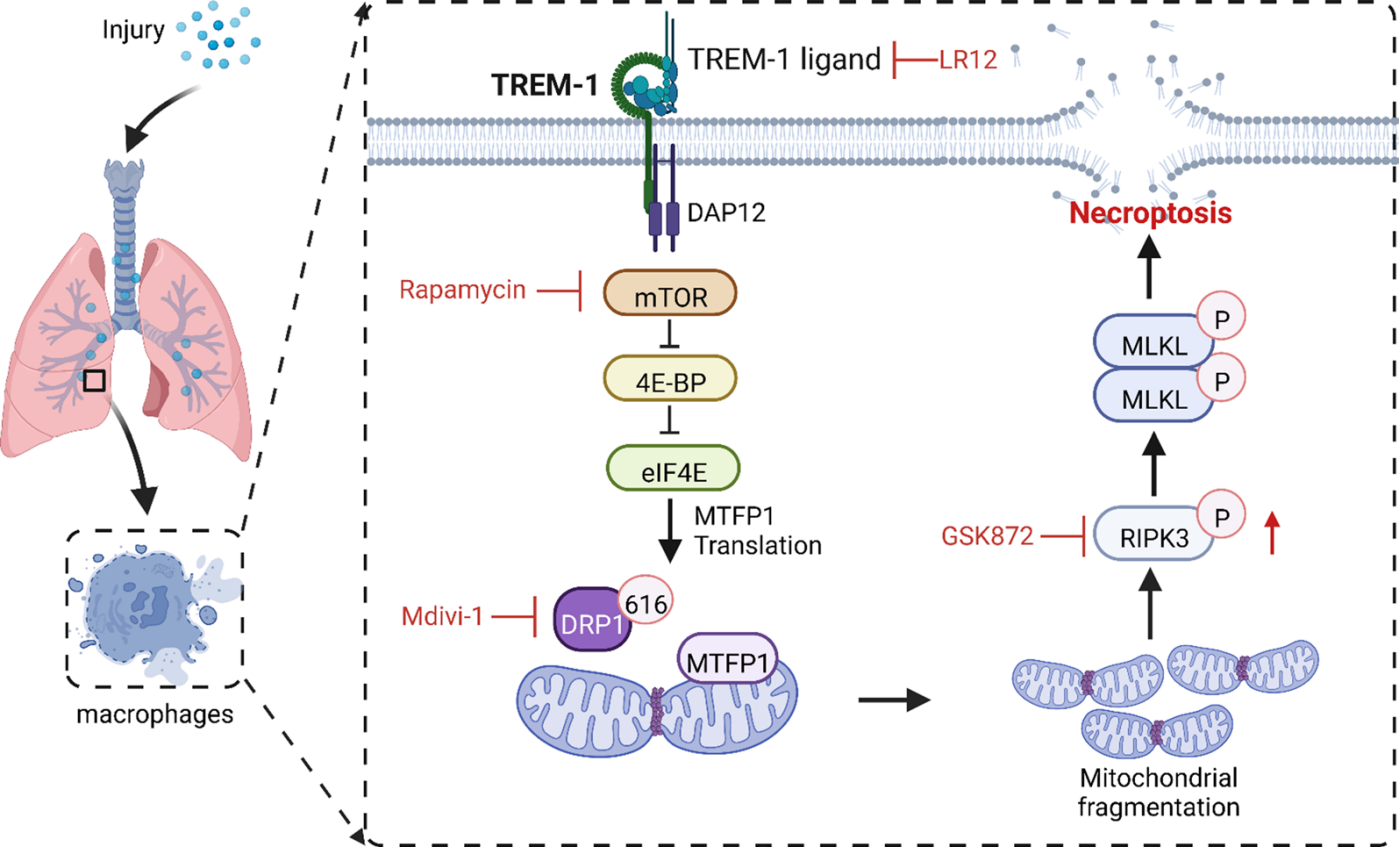
Schematic illustration. TREM-1 activation induces necroptosis via the mTOR-dependent mitochondrial fission pathway in macrophages

Inflammation is critical in the host’s defensive mechanism against microbial invasion. Excessive inflammation has the potential to inflict significant damage to cells and lung tissue. Necroptosis of macrophages occupies a vital position in exacerbating pulmonary inflammation in ALI [7]. However, the molecular mechanism that triggers macrophage necroptosis is still unclear. Herein, we revealed that TREM-1 induced necroptosis in AlvMs, a novel mechanism triggering inflammatory cascade response to exacerbate ALI. Mechanistically, we identified that TREM-1 activation recruited DRP1 to drive mitochondrial fission via mTOR signaling, impairing mitochondrial dynamics and finally triggering necroptosis of macrophages (Fig. 7). Together with our findings, this supports that TREM-1-induced necroptosis of macrophages is a fundamental mechanism in ALI.

Our study identified TREM-1 as the necroptotic stimulus for macrophages during ALI. Apoptosis, pyroptosis, ferroptosis, and necroptosis are the prominent regulated cell deaths in macrophages during ALI [9]. In contrast to non-lytic and usually non-immunogenic apoptosis, ferroptosis, pyroptosis, and necroptosis are lytic and highly inflammatory [43]. It is increasingly recognized that inflammation and immunogenic macrophage death reciprocally affect each other, creating an auto-amplifying loop of these two factors, which in turn exaggerates inflammation [9, 44]. Therefore, pharmacological manipulation of the macrophages’ immunogenic death signaling may potentially serve as a reasonable therapeutic strategy for ALI. We found that TREM-1 blockade with LR12 attenuated necroptosis of macrophages in LPS-induced ALI in mice. Moreover, TREM-1 activation increased inflammatory necroptosis in macrophages. The necroptosis pathway requires RIPK3 to be activated, causing MLKL to be phosphorylated and oligomerized, then translocating to the plasma membrane to form pores that break down the membrane and lead to cell lysis [45, 46]. Here, we showed that TREM-1 initiated necroptosis of macrophages, which is dependent on RIPK3 kinase activity. It has been reported that TREM-1 activation may induce pyroptosis. TREM-1 exacerbates neuroinflammatory injury via NLRP3 inflammasome-mediated pyroptosis [47]. However, GSDMD-dependent pyroptosis was not altered by agonist anti-TREM-1 Ab (Mab1187). We suggest that this may be due to the low expression of TREM-1 on non-microbial inflammatory macrophages [16], as the low TREM-1 activation using the Mab1187 is insufficient to induce macrophage pyroptosis. Another study shows that MLKL activation in monocytes and bone marrow-derived macrophages (BMDMs) also activates the NLRP3 inflammasome and caspase-1-dependent but GSDMD-independent IL-1β processing and release besides necroptosis [48]. Our study supports that TREM-1 activation triggers necroptosis of macrophages, promoting an inflammatory cascade amplification response, ultimately exacerbating lung injury.

We found that MLKL is expressed in particular AlvMs, and blockade of TREM-1 attenuates MLKL^+^ AlvMs. The distinct pulmonary resident macrophage subsets have been described in the murine lung, for example, SiglecF^hi^ CD11c^hi^ AlvMs and SiglecF^lo^ CD11b^hi^ IntMs [38]. AlvMs are derived from fetal liver monocytes and populate the alveolar spaces in the lung after birth. Their primary physiological function is the clearance of the constantly renewed pulmonary surfactant. They also have the function of phagocytosing inhaled particles and performing immune surveillance [49]. Our results found that AlvMs are more susceptible to stimulation and undergo necroptosis. The necroptosis of AlvMs disrupts the cellular membrane and releases cell debris or DAMPs, which activates other immune cell populations and forms a positive feedback loop, exacerbating ALI. IntMs are derived from blood monocytes. Their predominant function is immune surveillance in the lung [50]. Our results showed no altered levels of MLKL are found in IntMs (SiglecF^lo^ CD11b^hi^) in ALI, which suggests that necroptosis occurs in particular macrophage subtypes. The precise localization of AlvMs populations is unclear. Lung macrophage populations undergo specific changes in the context of distinct stress [51]. In future studies, we will use single-cell mRNA sequencing to identify independent populations exhibiting distinct gene expression profiles and macrophage phenotypes in ALI. On this basis, the differences and significance of MLKL levels in different macrophage populations were further discussed.

Mitochondria are highly dynamic, double-membrane organelles that are essential for the survival and functions of eukaryotic organisms [41]. Mitochondrial dynamics involve mitochondrial fusion and fission [52]. Excessive mitochondrial fission is an early marker of mitochondrial damage that leads to energy shortage and ultimately cell death [53]. In this study, we found that the mitochondria of TREM-1-activated macrophages show an over-divided morphology. Inhibition of mitochondrial fission could reverse the TREM-1-induced necroptosis, but not apoptosis, in macrophages. This suggests that TREM-1-induced mitochondrial fission specifically triggers necroptosis, but not apoptosis, in macrophages. DRP1-dependent mitochondrial fission has been proposed to be a prerequisite for mitophagy [53]. DRP1-mediated excessive mitochondrial fission induces LC3B lipidation and mitophagy, which requires Pink1 [54]. Here, we found that TREM-1 activation upregulated mitophagy-related proteins. Mdivi-1, a selective DRP1 inhibitor, simultaneously attenuated the TREM-1-induced mitophagy. Our previous studies showed that treatment with Mdivi-1 significantly reduced mitophagy in MLE12 cells following shRNA-mediated silencing of Idh3a and Slc25a1 [14]. Others have shown that inhibiting mitochondrial fission through DRP1(K38A) or FIS1 RNAi decreased mitophagy [55]. Although the mitochondrial network appears to be homogenous, fission can produce the segregation of damaged mitochondrial components with a reduced mitochondrial membrane potential. Daughter mitochondria are less likely to re-fuse with the mitochondrial network and are more likely to be degraded by mitophagy [56]. Therefore, mitochondrial fission is considered essential for mitophagy.

Although mTOR has been previously linked to macrophage polarization and cell migration [57], we discovered an unexpected role for mTOR in macrophage necroptosis. Others have shown that mTOR counteracts TRIM11-mediated ubiquitination and degradation of RIPK3, leading to epithelial necroptosis [58]. mTOR inhibition protects cardiomyocytes from necroptosis by a TFEB-dependent mechanism [59]. Overall, studies from others and ous strongly suggest that precise control of the mTOR activity is crucial for macrophage function and survival. Mechanistically, we found that TREM-1 activated mTOR signaling to promote DRP1-dependent mitochondrial fission. Excessive activation of mTOR reportedly induces phosphorylation of Drp1, resulting in mitochondrial fragmentation in NK cells [60]. mTOR stimulates mitochondrial fission via 4E-BP-mediated translational regulation of the mitochondrial fission factor, MTFP1 [57]. MTFP1 is an integral protein of the mitochondrial intermembrane whose loss results in a hypoperfused mitochondrial reticulum, whereas its overexpression engenders fragmentation [42, 61]. We observed a concomitant elevation of mTOR and MTFP1 in TREM-1-activated macrophages. Inhibition of mTOR reverses the TREM-1-induced excessive mitochondrial fission and necroptosis in macrophages. PGAM5 is a mitochondrial protein phosphatase that initiates mitochondrial fission by dephosphorylating DRP1^S637^ and promoting the translocation of DRP1 to the mitochondria [62]. PGAM5 is defined as the convergent point for multiple necroptosis pathways [63]. PGAM5 and its downstream Drp1-mediated mitochondrial fission are obligatory steps that drive the execution of hepatic necroptosis and tissue damage [64]. Here, our results found that TREM-1 activation promotes the expression of PGAM5, and inhibition of mTOR reverses TREM-1-induced PGAM5, suggesting the mTOR/PGAM5 axis may play an essential role in TREM-1-induced necroptosis. Overall, our studies strongly suggest that precise control of the mTOR activity is crucial for mitochondrial dynamics.

There are still some limitations in this study. Our work identified TREM-1 as a necroptotic stimulus of macrophages, while we did not assess the markers of ferroptosis in TREM-1-induced macrophages. TREM-1’s impact on various forms of macrophage death [65, 66], including newly identified necroptosis, implicates its diverse role in macrophage death. As such, TREM-1-mediated macrophage death is not strictly limited to a specific modality but is mediated through several pathways. RIPK3 inhibitor largely blocked TREM-1-induced macrophage death, so we hypothesize that TREM-1 may mainly induce macrophage necroptosis. In future studies, it is necessary to assess the presence of various cell death markers/pathways in TREM-1-activated macrophages to ascertain if a particular pathway is activated or is intertwined with other mechanisms to induce cell death. This may also uncover the predominance of a specific cell death pathway in macrophages during TREM-1 stimulation. In addition, this study focused on the molecular mechanism of mitochondrial fission-induced macrophage necroptosis. The specific mechanisms by which TREM-1-induced mitochondrial fission leads to mitophagy and necroptosis were not fully recognized, so ongoing efforts are required to explore the specific molecular mechanism. Single-cell RNA sequencing can provide more informative and accurate clues [67].

## Conclusion

In summary, our studies identified TREM-1 as the necroptotic stimulus of AlvMs in ALI. TREM-1 activates mTOR signaling, promotes transcription of MTFP1 and PGAM5, etc., and induces mitochondrial fission in macrophages. We also provide compelling evidence suggesting that mTOR-dependent mitochondrial fission is the underpinning of TREM-1-triggered necroptosis and inflammation. This study provides a practicable idea for ALI drug therapy targeting TREM-1 in the future.

## Supplementary Information

**Additional file 1: Figure S1**. The expression of MLKL was not altered in IntMs (SiglecF^lo^ CD11b^hi^) in ALI. A, Flow cytometry analysis of MLKL^+^ IntMs, *n* = 3. **Figure S2**. Mitochondrial membrane potential is dissipated by the treatment with Mab1187. Macrophages were incubated with plate-bound isotype-matched control or plate-bound anti-TREM-1 mAb (10 μg/mL). A-B, Twenty-four hours later, representative images of macrophages loaded with the mitochondrial membrane potential indicator JC‐1 (bar = 50 μm) and quantification of mitochondrial membrane potential were analyzed by ImageJ, *n* = 3. **Figure S3**. Mdivi-1 simultaneously attenuated TREM-1-induced mitophagy. Macrophages were premixed with PBS control or Midvi-1 (100 nM) before incubating with plate-bound agonistic anti-TREM-1 mAb. A-B, the protein of Pink1, Beclin1, and LC3II protein in cell lysate. *n* = 3 biological replicates. **Figure S4**. TREM-1 induces apoptosis of macrophages independent of mitochondria fission. A-D, the protein of caspase-6, pro-caspase-6, caspase-3, and pro-caspase-3. E–G, pro-GSDMD, and mature-GSDMD protein in cell lysate. *n* = 3 biological replicates. **Figure S5**. Inhibition of mTOR showed a partial restoration in mitochondrial membrane potential induced by TREM-1. A-B, macrophages were premixed with PBS control or rapamycin (10 nM) before incubating with plate-bound agonistic anti-TREM-1 mAb. Representative images of macrophages loaded with the mitochondrial membrane potential indicator JC‐1; bar = 50 μm.

## Data Availability

The datasets supporting the conclusions of this article are included within the article and its additional file. For any further data requests, please contact the corresponding author.

## Abbreviations

4E-BPs: Translation initiation factor 4E (eIF4E)-binding proteins
ALI: Acute lung injury
AlvMs: Alveolar macrophages
DAP12: The adaptor protein 12-kDa DNAX activating protein
DAMPs: Damage-associated molecular patterns
DRP1: GTPase dynamin-related protein 1
ELISA: Enzyme-linked immunosorbent assay
GSDMD: Gasdermin D
H&E: Hematoxylin and eosin
IL-1β: Interleukin-1β
IntMs: Interstitial macrophages
LPS: Lipopolysaccharide
MLKL: Mixed lineage kinase domain-like pseudokinase
mTOR: The nutrient sensor mechanistic target of rapamycin complex
MFN2: Mitofusin2
MFF: Mitochondrial anchor’s mitochondrial fission factor
MTFP1: Mitochondrial fission process protein 1
OPA1: Optic atrophy1
PAMPs: Pathogen-associated molecular patterns
PBS: Phosphate-buffered saline
PGAM5: Phosphatase phosphoglycerate mutase family member 5
RIPK3: Receptor-interacting serine/threonine-protein kinase 3
Pink1: The phosphatase, and tensin homolog-induced kinase 1
RSV: Human respiratory syncytial virus
TREM-1: Triggering receptor expressed on myeloid cells-1
TNF-α: Tumor necrosis factor-alpha
