# Supplementary material for: TREM-1 triggers necroptosis of macrophages through mTOR-dependent mitochondrial fission during acute lung injury
Source: J Transl Med. 2023 Mar 6;21:179. doi: 10.1186/s12967-023-04027-4 (PMC9990355; doi:10.1186/s12967-023-04027-4)

**Additional material**

**
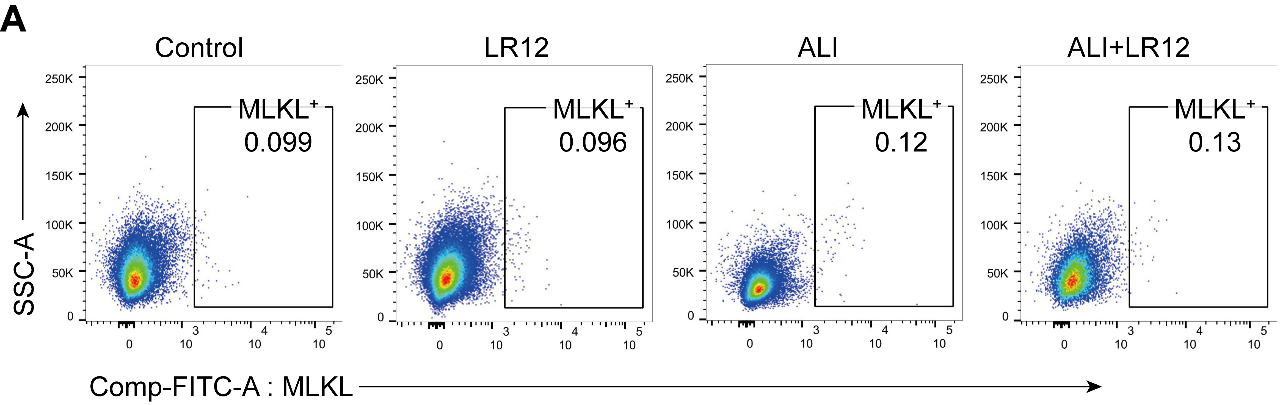
**

**Figure S1**. **The expression of MLKL was not altered in IntMs (SiglecF^lo^ CD11b^hi^) in ALI.** A, Flow cytometry analysis of MLKL^+^ IntMs, *n*=3.

**
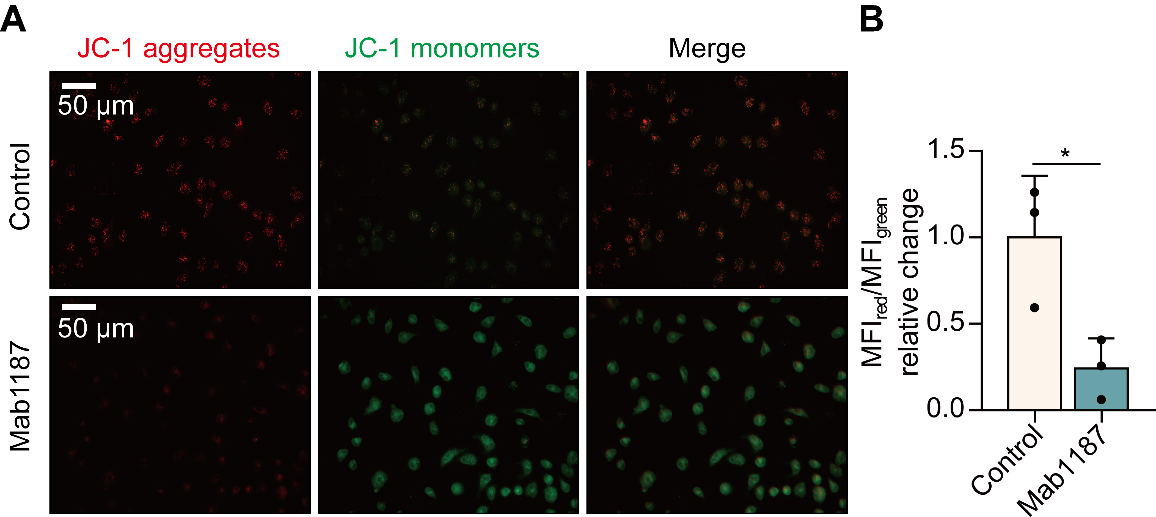
**

**Figure S2**. **Mitochondrial membrane potential is dissipated by the treatment with Mab1187.** Macrophages were incubated with plate-bound isotype-matched control or plate-bound anti-TREM-1 mAb (10 μg/mL). A-B, Twenty-four hours later, representative images of macrophages loaded with the mitochondrial membrane potential indicator JC‐1 (bar=50 μm) and quantification of mitochondrial membrane potential were analyzed by ImageJ, *n*=3.


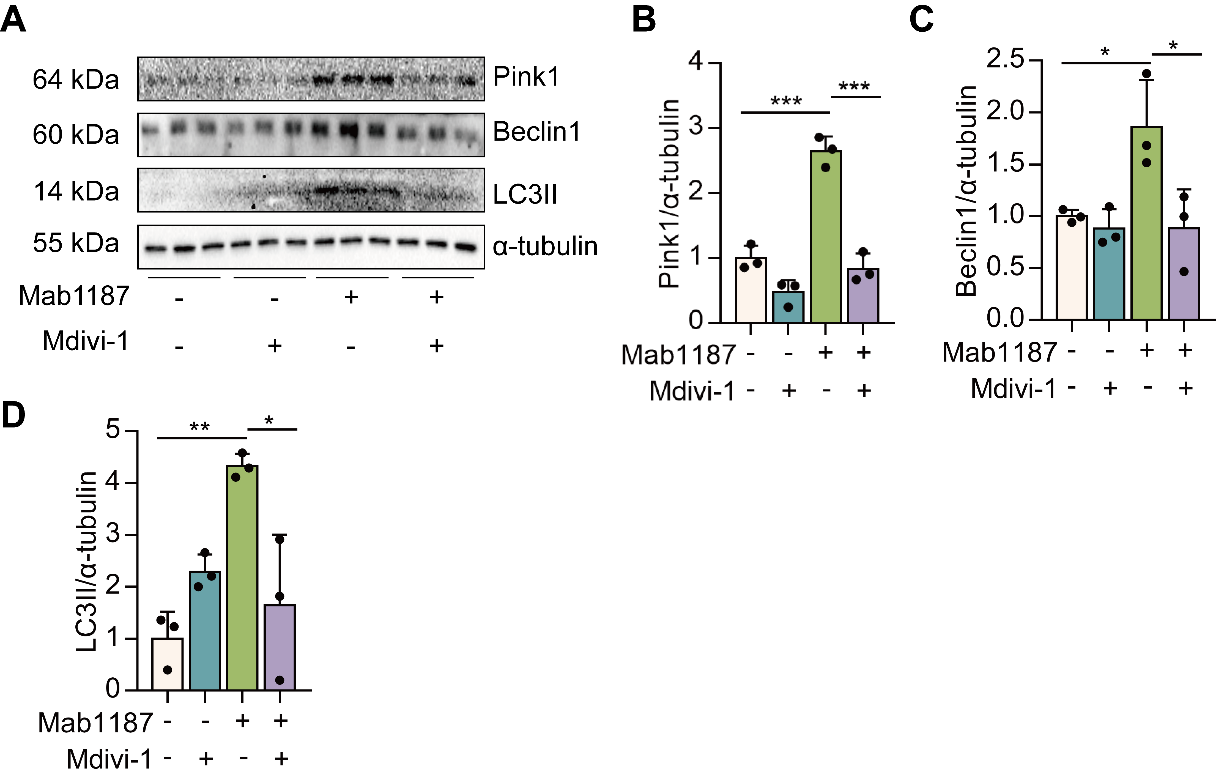


**Figure S3**. **Mdivi-1 simultaneously attenuated TREM-1-induced mitophagy.** Macrophages were premixed with PBS control or Midvi-1 (100 nM) before incubating with plate-bound agonistic anti-TREM-1 mAb. A-B, the protein of Pink1, Beclin1, and LC3Ⅱ protein in cell lysate. *n*=3 biological replicates.


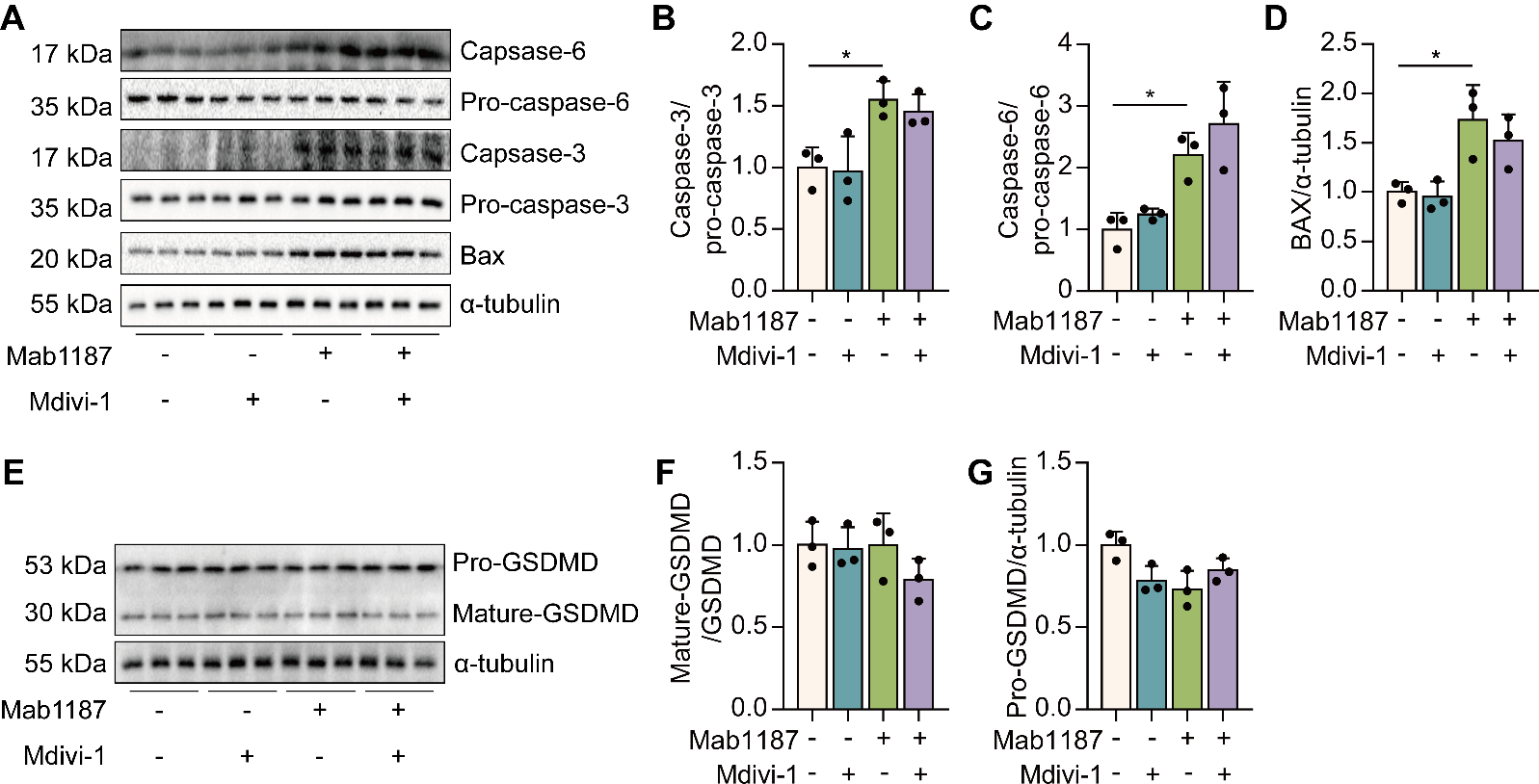


**Figure S4**. **TREM-1 induces apoptosis of macrophages independent of mitochondria fission.** A-D, the protein of caspase-6, pro-caspase-6, caspase-3, and pro-caspase-3. E-G, pro-GSDMD, and mature-GSDMD protein in cell lysate. *n*=3 biological replicates.


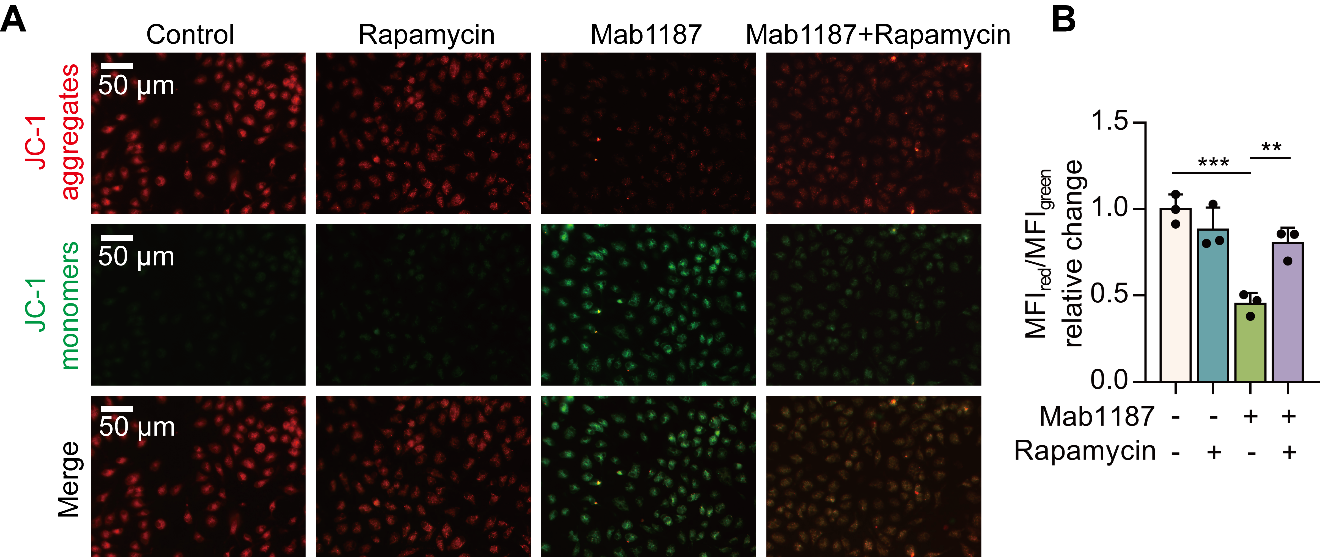


**Figure S5**. **Inhibition of mTOR showed a partial restoration in mitochondrial membrane potential induced by TREM-1.** A-B, macrophages were premixed with PBS control or rapamycin (10 nM) before incubating with plate-bound agonistic anti-TREM-1 mAb. Representative images of macrophages loaded with the mitochondrial membrane potential indicator JC‐1; bar=50 μm.


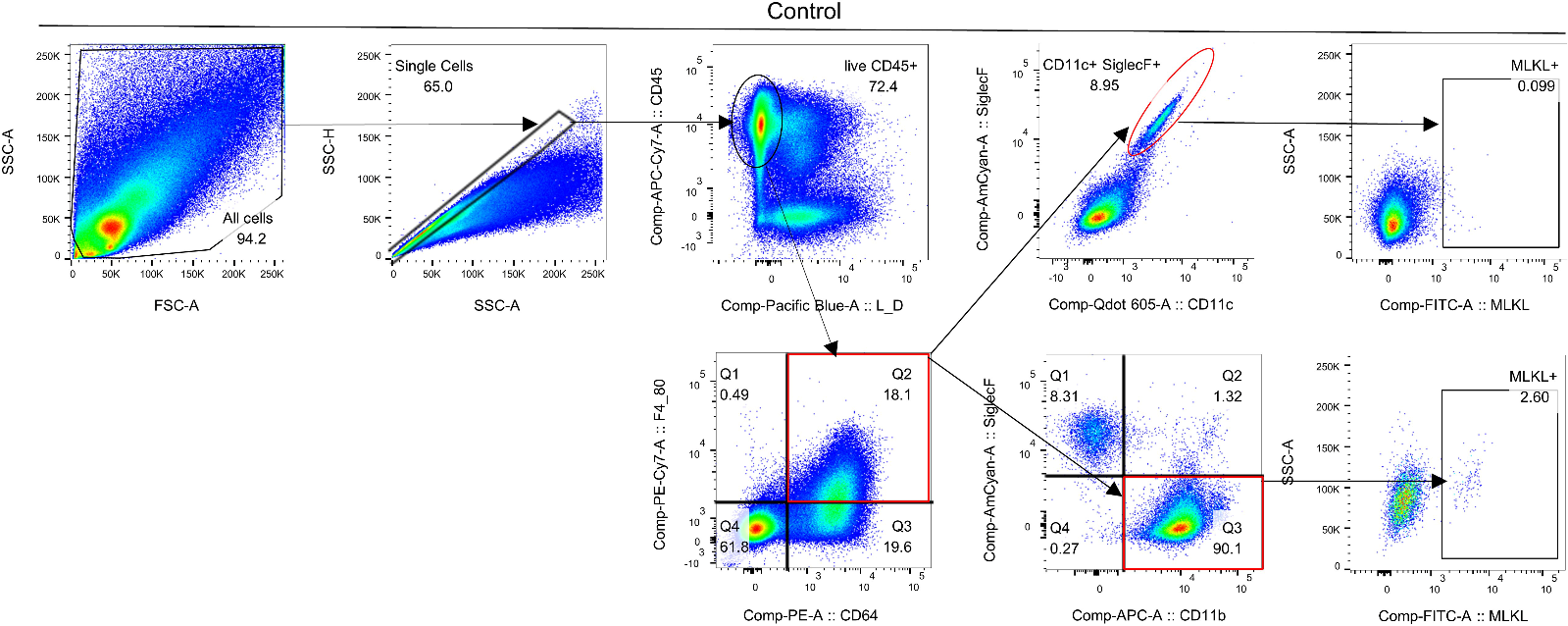


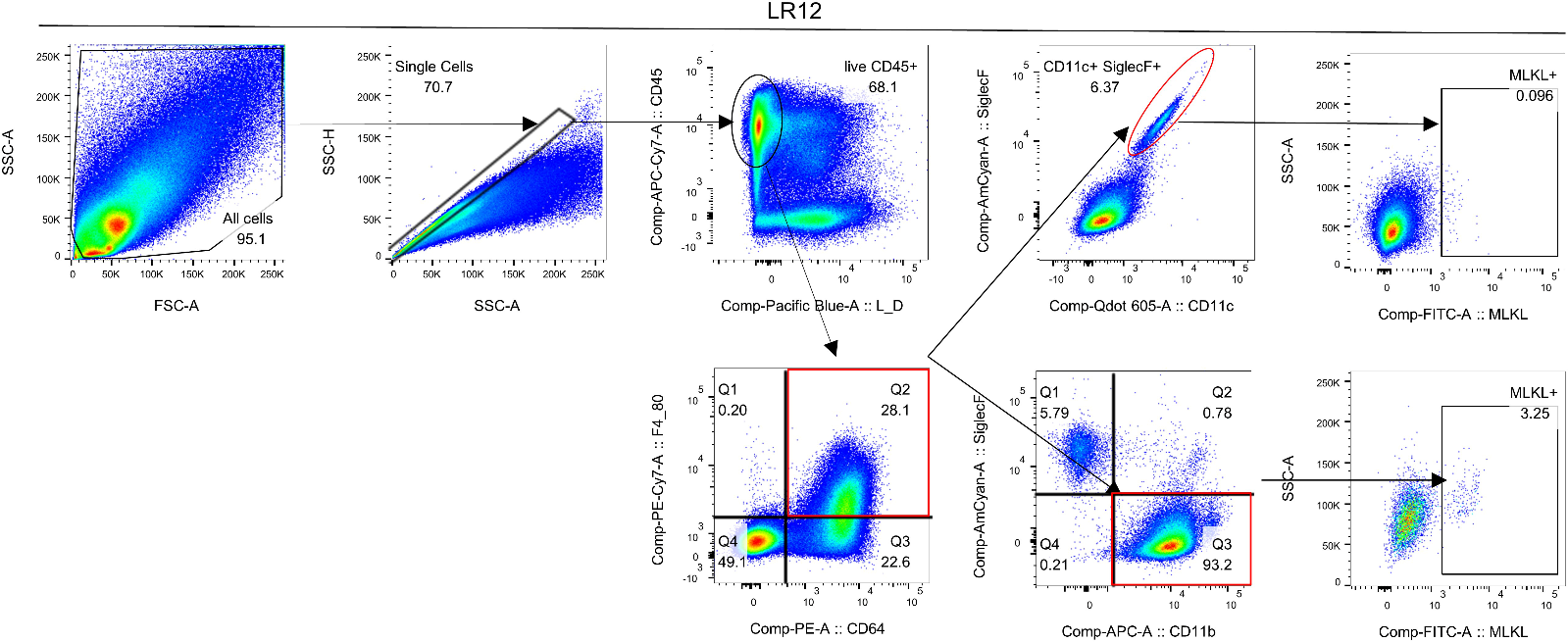


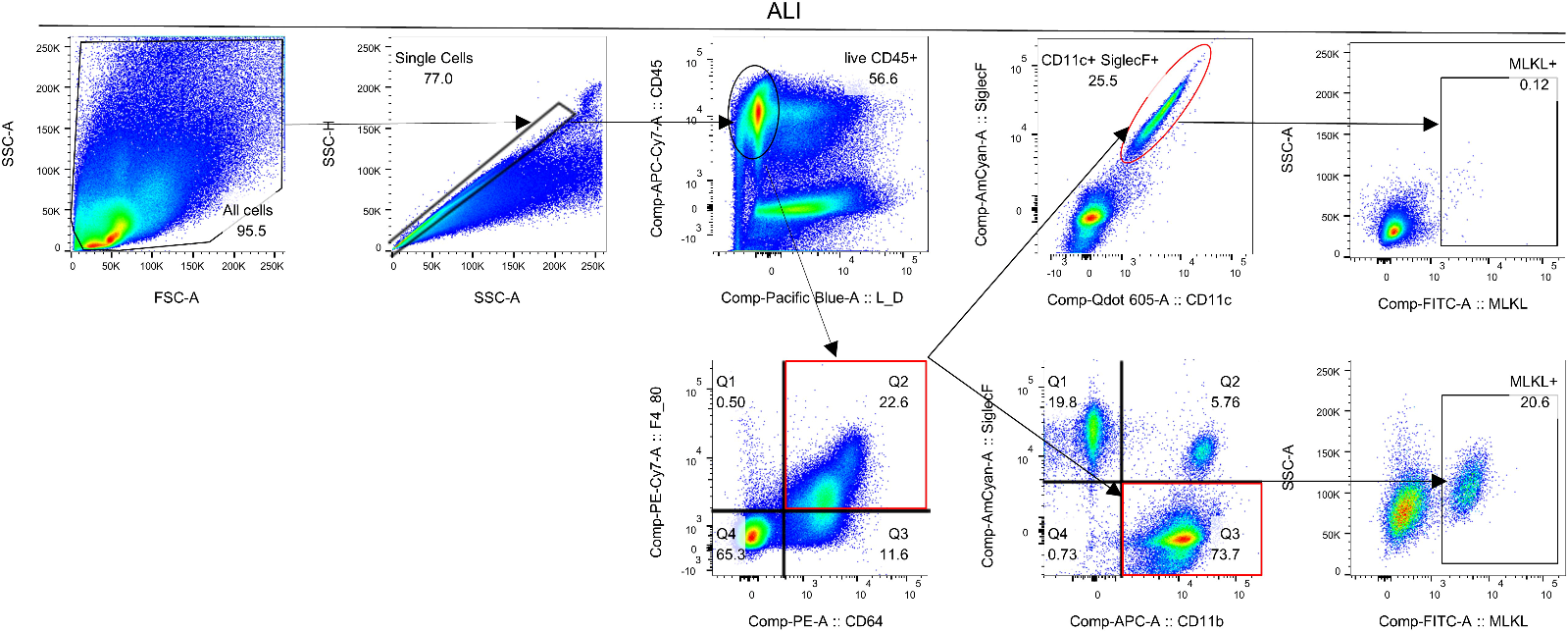


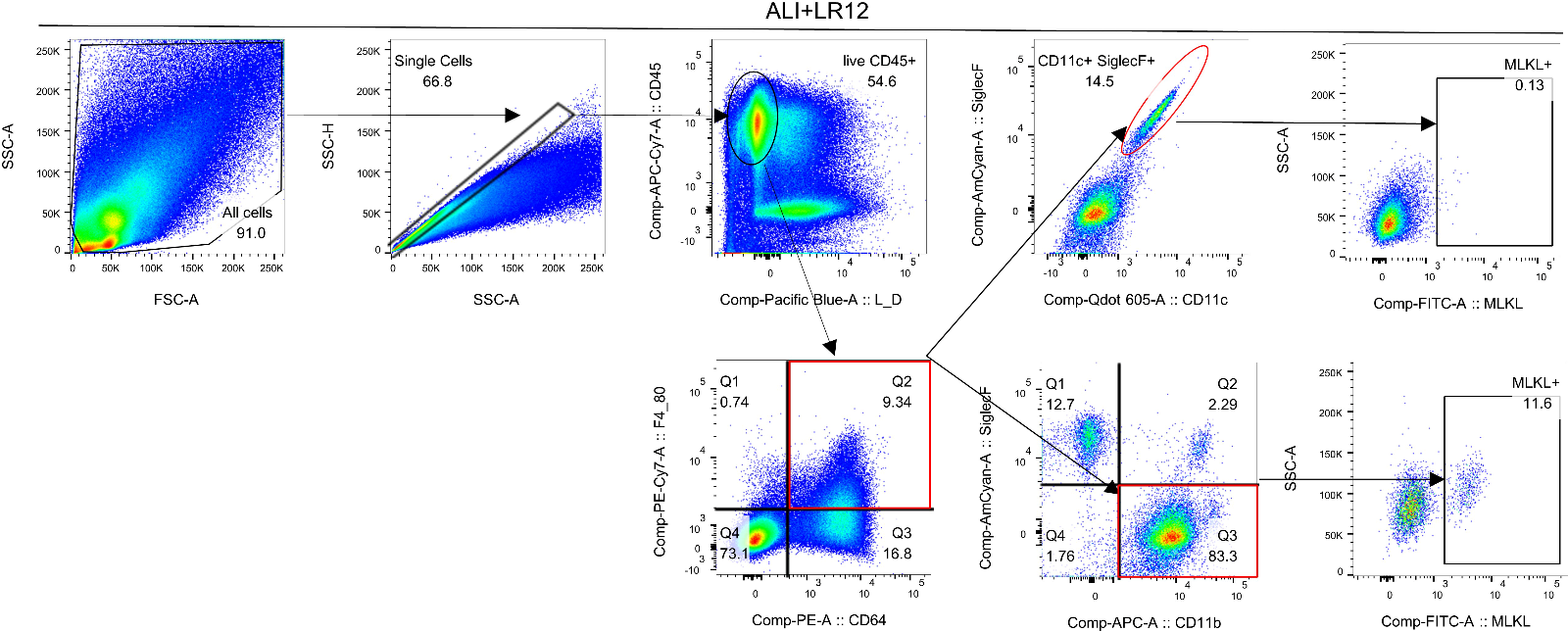

Supplement: Supplementary file 1 — Additional file 1: Figure S1. The expression of MLKL was not altered in IntMs (SiglecFlo CD11bhi) in ALI. A, Flow cytometry analysis of MLKL+ IntMs, n = 3. Figure S2. Mitochondrial membrane potential is dissipated by the treatment with Mab1187. Macrophages were incubated with plate-bound isotype-matched control or plate-bound anti-TREM-1 mAb (10 μg/mL). A-B, Twenty-four hours later, representative images of macrophages loaded with the mitochondrial membrane potential indicator JC‐1 (bar = 50 μm) and quantification of mitochondrial membrane potential were analyzed by ImageJ, n = 3. Figure S3. Mdivi-1 simultaneously attenuated TREM-1-induced mitophagy. Macrophages were premixed with PBS control or Midvi-1 (100 nM) before incubating with plate-bound agonistic anti-TREM-1 mAb. A-B, the protein of Pink1, Beclin1, and LC3II protein in cell lysate. n = 3 biological replicates. Figure S4. TREM-1 induces apoptosis of macrophages independent of mitochondria fission. A-D, the protein of caspase-6, pro-caspase-6, caspase-3, and pro-caspase-3. E–G, pro-GSDMD, and mature-GSDMD protein in cell lysate. n = 3 biological replicates. Figure S5. Inhibition of mTOR showed a partial restoration in mitochondrial membrane potential induced by TREM-1. A-B, macrophages were premixed with PBS control or rapamycin (10 nM) before incubating with plate-bound agonistic anti-TREM-1 mAb. Representative images of macrophages loaded with the mitochondrial membrane potential indicator JC‐1; bar = 50 μm. [file 12967_2023_4027_MOESM1_ESM.docx]
